# Are reduced internal nasal dimensions a risk factor for obstructive sleep apnea syndrome?

**DOI:** 10.1016/j.bjorl.2020.06.014

**Published:** 2020-07-30

**Authors:** Sergio Henrique Kiemle Trindade, Inge Elly Kiemle Trindade, Andressa Sharllene Carneiro da Silva, Bruna Mara Adorno Marmontel Araújo, Ivy Kiemle Trindade-Suedam, Ana Claudia Martins Sampaio-Teixeira, Silke Anna Theresa Weber

**Affiliations:** a Universidade de São Paulo (USP), Faculdade de Odontologia de Bauru, Bauru, SP, Brazil; b Universidade de São Paulo (USP), Hospital de Reabilitação de Anomalias Craniofaciais (HRAC), Unidade de Estudos do Sono do Laboratório de Fisiologia, Bauru, SP, Brazil; c Universidade de São Paulo (USP), Hospital de Reabilitação de Anomalias Craniofaciais (HRAC), Seção de Otorrinolaringologia, Bauru, SP, Brazil; d Hospital Estadual de Bauru, Divisão de Otorrinolaringologia, Bauru, SP, Brazil; e Universidade Estadual Paulista (UNESP), Faculdade de Medicina de Botucatu, Botucatu, SP, Brazil; f Universidade Estadual Paulista (UNESP), Hospital das Clínicas da Faculdade Medicina de Botucatu, Laboratório de Diagnóstico e Tratamento dos Distúrbios Respiratórios do Sono, Botucatu, SP, Brazil

**Keywords:** Nasal obstruction, Acoustic rhinometry, Sleep apnea

## Abstract

**Introduction:**

Obstructive sleep apnea syndrome is a high-prevalence disorder found in the population. Studies have shown a possible association between nasal obstruction and obstructive sleep apnea syndrome, but the existence of a association between the degree of nasal obstruction and obstructive sleep apnea syndrome severity has not yet been proven.

**Objective:**

To evaluate the internal nasal dimensions of adults with primary snoring and obstructive sleep apnea syndrome by acoustic rhinometry and to correlate the findings with obstructive sleep apnea severity.

**Methods:**

Twenty-one male Caucasian subjects with complaints of snoring and/or respiratory pauses during sleep, aged between 18 and 60 years of age, were evaluated. After clinical evaluation, otorhinolaryngological examination and flexible nasopharyngolaryngoscopy, all patients underwent type III polysomnography. The participants were divided into two groups according to symptom severity: group 1, primary snoring and/or mild obstructive sleep apnea syndrome(n = 9) and group 2, moderate/severe obstructive sleep apnea syndrome (n = 12). Internal nasal dimensions were measured by acoustic rhinometry, analyzing minimum cross sectional area (CSA) and three nasal segment volumes.

**Results:**

The respiratory event index corresponded to 8.1 ± 4.0 in group 1 and 47.5 ± 19.1 in group 2. In group 1, the cross-sectional areas values, in cm2, corresponded to: CSA 1 = 1.1 ± 0.4; CSA 2 = 2.1 ± 0.9; CSA 3 = 3.5 ± 1.8 and, in group 2: CSA 1 = 1.2 ± 0.3, CSA 2 = 2.0 ± 0.5; CSA 3 = 2.8 ± 0.7. In group 1, volumes (V), in cm3, corresponded to: V1 = 3.5 ± 1.0; V2 = 9.3 ± 5.0; V3 = 40.2 ± 21.5 and in group 2 a: V1 = 3.6 ± 0.5; V2 = 7.6 ± 1.5; V3 = 31.5 ± 6.7. Cross-sectional area and volume ​​did not differ between groups.

**Conclusion:**

There were no significant differences in the cross-sectional areas and nasal volumes between individuals with primary snoring-mild obstructive sleep apnea syndrome and moderate-severe obstructive sleep apnea syndrome. Differently to the raised hypothesis, our results suggest that there is no association between internal nasal dimensions and severity of obstructive sleep apnea syndrome.

## Introduction

Obstructive sleep apnea syndrome (OSAS) is a high-prevalence disorder among the general population. Research studies, such as the Sleep Heart Health Study, showed that 24% of the evaluated men and 9% of women had moderate OSAS.^1^ In turn, the Wisconsin Sleep Study Cohort showed an increase in the prevalence of OSAS in different age groups: 10% of men and 3% of women in the 30 to 49 age group, and 17% of men and 9% of women aged 50 to 70 years.^2^ More recently, a study carried out in Switzerland, showed that 50% of evaluated men and 23% of women had moderate OSAS.^3^ In Brazil, a study that assessed the prevalence of OSAS in the population of the city of São Paulo, observed that 32.8% of the adult population had polysomnographic criteria compatible with the diagnosis of OSAS.^4^ It is noteworthy that, despite its high prevalence, OSAS is still underdiagnosed. In the United States of America, it is estimated that 82% of men and 93% of women with OSAS are not acurately diagnosed.^5^

Patients with OSAS typically have excessive daytime sleepiness, decreased cognitive performance, reduced quality of life and increased risk of developing cardiovascular disease.^6^, ^7^, ^8^ Age over 60 years, male gender, obesity, craniofacial dysmorphisms and hypothyroidism are well-established risk factors for OSAS.^1^, ^5^, ^9^, ^10^

Studies have also suggested a possible association between nasal obstruction and OSAS, attributing the genesis of OSAS to the turbulent air flow resulting from nasal obstruction in different segments. As the nasal cavities are the gateway to the upper airways, obstructions resulting from nasal pathologies, such as septal deviation and nasal turbinate hypertrophy can theoretically contribute to the generation of turbulent air flow, consequently triggering OSAS.^11^, ^12^, ^13^, ^14^ Theoretically, the upper airways behave similarly to a Starling resistor, where the collapsible segment is the pharynx and the two rigid ends are the nasal cavities and the larynx. The greater the resistance to airflow in the fixed segments (nasal cavity and larynx) and the greater the flaccidity of the flexible segment (pharynx), the greater the collapse of the collapsible segment.^15^, ^16^ Thus, the association between nasal obstruction and OSAS gains biological plausibility.

A study demonstrated that individuals with chronic nasal obstruction have greater daytime sleepiness, resulting from sleep fragmentation, generated by multiple micro-awakenings and that the treatment of nasal obstruction significantly improved daytime symptoms.^17^ In theory, these findings reinforce the evidence that the degree of nasal patency plays an important role in OSAS and its associated symptoms.

The evaluation of nasal patency has historically been based on clinical impressions, data from anamnesis and visual inspection through rhinoscopy and/or nasofibroscopy. In recent years, however, acoustic rhinometry, introduced by Sondhi and Gopinath,^18^ has been used as an objective assessment of internal nasal dimensions that, ultimately, reflect the degree of nasal patency. In brief, the sound signals generated by the rhinometer are reflected by intranasal and nasopharyngeal structures, being captured and analyzed using a software that calculates different variables, which correspond to minimum cross-sectional areas and volumes in different segments. Currently, the importance of acoustic rhinometry is widely recognized as a specific test of internal nasal dimensions.^19^, ^20^, ^21^, ^22^, ^23^, ^24^, ^25^, ^26^, ^27^ Subnormal values ​​have been observed in individuals with nasal obstruction of different etiologies. 28, 29, 30, 31

Although recent studies have contributed to the understanding of the association between nasal obstruction and OSAS,^10^, ^11^, ^12^, ^13^, ^14^ several aspects still remain controversial, particularly because methodological limitations impairs comparison between studies. Briefly, it seems that the influence of nasal obstruction on OSAS cannot yet be established with the level of scientific evidence available to date. Therefore, this study aimed to assess the internal nasal dimensions of adults with primary snoring and mild OSAS, compared to individuals with moderate and severe OSAS, using acoustic rhinometry.

## Methods

This study is derived from a postdoctoral research and was approved by the Human Research Ethics Committee of Faculdade de Medicina de Botucatu - Unesp, under number 4393-2012 and was carried out after the Free and Informed Consent form (FICF) was signed by the participant.

Adult male Caucasian individuals, aged between 18 and 60 years, regularly followed at the Otorhinolaryngology Outpatient Clinic of Hospital Estadual de Bauru (HEB), who had snoring or respiratory pauses as the main complaint during sleep were selected.

All participants were clinically evaluated, focusing on complaints compatible with OSAS, and were submitted to a physical examination with nasoendoscopic assessment of the nasal and pharyngeal anatomy. The examination aimed to classify septal deviations and rule out obstructive upper airway lesions, which are difficult to visualize at the otorhinolaryngological physical examination. The degree of lower turbinate hypertrophy and septal deviations was assessed visually by the main investigator. Septal deviations were categorized as follows: presence or absence; predominant laterality (right or left); predominant location (anterior, when it affected the membranous and/or cartilaginous septum, and posterior, when it predominantly affected the transition between the cartilaginous and bony septa, perpendicular lamina of the ethmoid bone and/or the vomer). The intensity of septal deviation was classified as Grade 1, when it obstructed up to 25% of the nasal cavity affected by the deviation, Grade 2, when it obstructed between 25% −50%, Grade 3, between 50% −75% and Grade 4, above 75% of the lumen of the nasal cavity. The inferior turbinates were classified according to the presence or absence of hypertrophy and affected segments (head, body or tail). The degree of turbinate obstruction followed the same criteria for septal deviations.

Patients with a history of previous surgery and nasal fracture, presence of malignant or benign nasal tumors, nasal polyps, craniofacial malformations, genetic syndromes, heart diseases, lung diseases and other clinical and mental conditions that did not allow all stages of the study to be performed, in addition to those who declined to sign the FICF form were not included from the study. Moreover, patients using topical nasal medications such as corticosteroids, mast-cell membrane stabilizers, topical vasoconstrictors, as well as systemic medications such as corticosteroids, antihistamines, leukotriene antagonists and antibiotics for treatment of rhinosinusopathies one month before the rhinometric analysis and polysomnography, were not included.

After clinical evaluation, the participants were referred for a type III polysomnography, using a Star Dust® polygraph (PhilipsRespironics), specifications.^32^ The examinations were carried out in the HEB wards. Respiratory sleep disorders, as well as respiratory events, were classified and recorded according to the American Academy of Sleep Medicine manual.^32^ After polysomnographies, the participants were allocated in two groups: G1 – Primary Snoring or mild OSA group; or G2 – Moderate or severe OSA group.

The patients were referred to undergo acoustic rhinometry at the Physiology Laboratory of *Hospital de Reabilitação de Anomalias Craniofaciais* - USP. Examination was performed in the morning of the day following polysomnography. For the rhinometric evaluation, the Eccovision Acoustic Rhinometer system (HOOD Laboratories) was used, according to protocols previously established by our laboratory. The parameters evaluated were: Cross Sectional Areas (CSA) and Volumes (V) in three different segments: CSA1 (area corresponding to the nasal valve), CSA2 (area corresponding to the anterior portion of the inferior nasal turbinate) and CSA3 (area corresponding to the tail of the inferior nasal turbinate), V1 (volume of the internal nasal segment between 10 to 32 mm from the nostril), V2 (volume of the segment located between 33 and 64 mm) and V3 (volume located after 70 mm). The examination was performed with the patient in the sitting position, with the chin and forehead supported by a frame specifically developed for this purpose, in order to keep the head stable during the examination.

In order to guarantee the reproducibility of the rhinometric curves, the equipment was calibrated at the beginning of each period of the day, according to the manufacturer's instructions, with the tube being positioned so as not to cause deformation of the nostril and, consequently, of the nasal valve. The measurements were always performed in the same room, in an environment with a relatively stable temperature (between 22° and 26 °C) and a controlled noise level (below 60 dB), after a period of patient adaptation to the environmental conditions of approximately 30 minutes, in the morning. The seal between the adapter and the nasal cavity was carried out using a neutral gel, aiming to avoid sound loss. The examination was performed during the voluntary suspension of nasal breathing, at the end of exhalation, and the patients were instructed to keep their mouth closed, without swallowing or moving their tongue at the time of data acquisition, which does not exceed 10 seconds, aiming to avoid interferences in measurements and the rhinogram quality.^23^, ^30^, ^31^, ^33^

### Sample size calculation and statistical analysis

For the formal sample size calculation (n), the minimum cross-sectional area of ​​the nasal cavity (CSA1 or minimum) corresponding to the nasal valve was chosen as the primary outcome, using normal values of adults with no evidence of nasal obstruction, same age range of form the Laboratory of Physiology of Hospital de Reabilitação de Anomalias Craniofaciais - USP.^33^ The results of Grymer et al.,^28^ were used, for the identification of nasal obstruction (CSA1 <  0.27cm^2^).

For the continuous variable CSA1, the possibility of a two-tailed alternative hypothesis was presumed, with an alpha of 5% and beta of 20% (power of 80%). Considering that the mean CSA1 value found by Gomes et al.^33^ was 0.54 ± 0.13 cm^2^ and assuming a clinically significant magnitude of effect as a 40% reduction in the CSA1 value, with a standard deviation from normal values ​​of approximately 20%, a standardized magnitude of effect equal to 2 was reached. For this magnitude and a standard deviation of ±0.13 cm^2^, the recommended number was 8 individuals in each experimental group.^34^

The *t* test for independent samples was used to compare data with normal distribution, whereas the Mann-Whitney test was used for data with non-parametric distribution. Values ​​of *p* <  0.05 were considered significant.

## Results

Table 1 shows the demographic characteristics, age, BMI (Body Mass Index) and the main results obtained. Despite older age and higher BMI in the moderate/severe OSAS group, this difference was not statistically significant.

**Table 1:** Demographic, polysomnographic and rhinometric data of individuals with primary snoring/mild OSAS and moderate/severe OSAS. The results are expressed as mean ± standard deviation.

Data	Variables	Primary snoring/mild OSAS (n = 9)	Moderate/severe OSAS (n = 11)
Demographic and anthropometric	Age	39 ± 11.2	47 ± 7.9
	BMI	28 ± 4.4	31.5 ± 4.4
Polysomnographic	REI	8.1 ± 4.0^a^	47.5 ± 19^a^
	OAI	2.0 ± 1.4^b^	32.8 ± 18^b^
	HI	5.6 ± 4.1	11.6 ± 9
	MD	19.7 ± 2.8^c^	24.2 ± 4^c^
	Mean SpO_2_	96 ± 1.4^d^	85 ± 10^d^
	ODI	9.4 ± 6.7^e^	45 ± 26^e^
Rhinometric (mean right and left nasal cavities)	CSA1	1.1 ± 0.4	1.2 ± 0.3
	CSA2	2.1 ± 0.9	2.0 ± 0.5
	CSA3	3.5 ± 1.8	2.8 ± 0.7
	V1	3.5 ± 1.0	3.6 ± 0.5
	V2	9.3 ± 5.0	7.6 ± 1.5
	V3	40.2 ± 21.5	35.1 ± 6.7

OSAS, Obstructive Sleep Apnea Syndrome; BMI, Body Mass Index; REI, Respiratory Events Index per hour; OAI, Obstructive Apnea Index per hour; HI, Hypopnea Index per hour; MD, Mean Duration in seconds of respiratory events; Mean SpO_2_, Mean Oxyhemoglobin Saturation; ODI, Oxyhemoglobin Desaturation Index per hour; CSA, Cross-sectional area; V, Volume; n, number of individuals.

* Equal letters indicate a statistically significant difference (*p* <  0.05).

Table 1 additionally shows the main polysomnographic and rhinometric results. Respiratory Events Index (REI)^32^ of the Primary Snoring group/mild OSAS (G1) was 8.1 ± 4 and in the moderate/severe OSAS Group (G2) it was 47.5 ± 19 events per hour, with the difference being statistically significant. The Obstructive Apnea Index (OAI) was 2.0 ± 1.4 and 32.8 ± 18 events per hour, respectively, with the difference also being statistically significant. The Hypopnea Index (HI) did not differ between the groups. The mean duration of obstructive apneas was significantly shorter in G1 (19.7 ± 2.8 s) than in G2 (24.2 ± 4 s). The same table also shows that the average O_2_ saturation was 96% ± 1.4% in the Primary Snoring/Mild OSAS group and 85%±10% in the Moderate/Severe OSA group and the desaturation index was 9.4 ± 6.7 and 45 ± 26 events per hour, respectively, with the differences observed between the two groups being statistically significant. The other data obtained in polygraphic examinations were incidental and were not considered for analysis in the present study.

For the analysis of rhinometric data (cross-sectional areas and volumes), the mean values ​of each parameter obtained in the right and left nasal cavities were calculated, in order to evaluate the nose as a whole, and not each nasal cavity separately. The mean values ​​of CSA in the Primary Snoring/mild OSAS group were (CSA1 = 1.1 ± 0.4; CSA2 = 2.1 ± 0.9; CSA3 = 3.5 ± 1.8 cm^2^) and in the moderate/severe OSAS group (CSA1 = 1.2 ± 0.3; CSA2 = 2.0 ± 0.5; CSA3 = 2.8 ± 0.7 cm2). Despite CSA3 being smaller in the moderate/severe OSAS group, differences were not statistically significant differences. The same happened for the mean nasal volumes in Group G1 (V1 = 3.5 ± 1.0; V2 = 9.3 ± 5.0; V3 = 40.2 ± 21.5 cm^3^) and in Group G2 (V1 = 3.6 ± 0.5; V2 = 7.6 ± 1.5; V3 = 31.5 ± 6.7 cm^3^).

## Discussion

The present study aimed to establish an association between internal nasal dimensions and OSAS severity. For this purpose, strict inclusion and exclusion criteria were adopted in order to obtain a homogeneous sample. Previous studies have shown that African descendants have larger nasal dimensions than Caucasians.^35^, ^36^ In turn, male individuals have larger internal nasal dimensions than females^33^ and adults have larger internal nasal dimensions than children and adolescents.^37^, ^38^ Thus, in order to avoid selection bias and increase the internal validity of the study, a convenience sample of male adult Caucasian individuals was selected and evaluated. Patients previously submitted to nasal surgery or with nasal fractures, polyps, tumors and other nasopharyngeal pathologies were not included in the sample. Additionally, individuals using topical nasal and systemic medications that could interfere with internal nasal dimensions were also excluded from the study. Therefore, the possibility of including patients with inflammatory nasal pathologies was minimized. Corroborating the homogeneity of the two samples, no significant differences were observed regarding age and BMI between the groups.

Regarding the OSAS severity criteria, it was observed that the moderate/severe OSAS group consistently showed significantly higher REI values, OAI and maximum apnea duration than the primary snoring/mild OSAS group. Moreover, oximetry-related parameters, such as mean saturation, were significantly lower in the moderate/severe OSAS group and the desaturation index was significantly higher. In brief, these results reinforce the patient selection homogeneity and show that the two groups represented the extremes of the disease spectrum. The use of other parameters such as Apnea-Hypopnea Desaturation Index (AHDI), which according to Otero et. al.^39^ has better sensitivity and specificity to quantify the intensity of OSAS, should be the focus of future studies. Since REI does not consider the time elapsed during respiratory events, as well as the intensity and duration of desaturation, AHDI could better represent the severity spectrum of OSAS.^39^ Likewise, Bosi M. et. al.^40^ showed that polysomnographic data other than REI can indicate different OSAS phenotypes.^40^ In light of these findings, new studies correlating different polysomnographic parameters with rhinometric data could provide additional valuable information on the pathophysiology of airways in OSAS and indicate clinically relevant parameters.

Acoustic rhinometry, a method that is well established in the literature, was used in the present study to assess the role of the proximal upper airway segment (nasal cavities) in the genesis of OSAS. Contrary to the initial hypothesis, that is, “the greater the severity of OSAS, the smaller the internal nasal dimensions”, there were no significant differences between the primary snoring/mild OSAS group and the moderate/severe OSAS group regarding all evaluated parameters (cross-sectional areas and volumes). It should be noted, however, that the data referring especially to CSA3, V2 and V3 point towards a tendency of smaller dimensions in the moderate/severe OSAS group, with reductions of about 20%, 18% and 22%, respectively, which should be explored in future studies. It is speculated that, with the increase in the sample size, these differences may become more evident. It is also important to note that the rhinometric analysis were performed in the sitting position, which is used routinely in laboratory tests, including all studies previously developed by the group, allowing comparisons with previously described results.^23^, ^30^, ^31^, ^33^^,^^38^ The influence of other positions, such as the supine position, will be the target of future investigations by our group.

Studies that evaluated nasal resistance using rhinomanometry, such as those by Blakely & Mohwald^41^ and Atkins et al.,^41^ in line with our findings, did not identify a correlation between resistance to nasal flow and OSAS severity. On the other hand, Lofaso et al.^11^ identified higher values ​​of nasal resistance in individuals with moderate/severe OSAS, when compared to the group with primary snoring. Using logistic regression, they identified that the increase in nasal resistance was an isolated risk factor for the genesis of OSAS. This divergent finding one may lie in the fact that nasal resistance is a flow-dependent measure, that is, it depends on the respiratory effort performed by the patient at the time of the measurement. This problem does not occur with acoustic rhinometry, as it is a static measure. Thus, the comparison of our results with the studies that used rhinomanometry is limited by the differences between the studied groups and the applied methodologies.

Recent studies that assessed the internal nasal dimensions of apneic patients using acoustic rhinometry showed divergent results. Rocha et al.^42^ identified smaller cross-sectional areas in the nasal valve region in the right nostril in patients with moderate and severe OSAS, with no differences in nasal volumes. Unlike the present study, the authors analyzed the nasal cavities independently and the study group predominantly consisted of women, which impairs comparison with our results. Vidigal et al.,^43^ with an experimental design similar to ours, except that the study group included men and women, also found no differences in the internal nasal dimensions between the groups with and without OSAS, measured by acoustic rhinometry.

Considering the present findings, the influence of internal nasal dimensions on the genesis of OSAS remains controversial. Our study indicates that reduced internal nasal dimensions have no influence on OSAS intensity. Future studies analyzing the differences between genders, ethnicities and that include a control group with no signs and symptoms of OSAS, confirmed by polysomnography, will be of great value. Additionally, undergoing studies in our laboratory, which evaluate nasal flow by computational fluid dynamics analysis and the impact of resistance to airflow of the fixed distal segment of the upper airway, the laryngeal block, referring to the model of the Starling resistor, will contribute to the elucidation of this complex question.

## Conclusion

The analysis carried out in the present study showed that primary snorers and individuals with mild OSAS, have similar internal nasal dimensions when compared to individuals with moderate-severe OSAS. The results, therefore, indicate a lack of association between reduced internal nasal dimensions and greater OSAS severity.

## Conflicts of interest

The authors declare no conflicts of interest.
